# Commissioning for infection prevention

**DOI:** 10.1186/2047-2994-4-S1-P267

**Published:** 2015-06-16

**Authors:** D Wright, Infection Prevention Society and Royal College of Nursing Commissioning Working Group

**Affiliations:** 1 Infection Prevention Society, Bathgate, UK

## Introduction

The changing face of healthcare commissioning within England provided Infection Prevention Specialists with an opportunity to embed Infection Prevention and Control (IPC) into the heart of contractual arrangements. The Infection Prevention Society (IPS) collaborated with the Royal College of Nursing (RCN) to help support members in meeting the challenge of ensuring this key element to achieve quality and safety is embedded in the emerging commissioning landscape

## Objectives

This paper will outline the work of the Commissioning Network in influencing IPC commissioning.

## Methods

A joint scoping day was with members of the IPS and RCN to gain an understanding of the implications to commissioning in IPC. Agreed outcomes included the immediate release of a joint position statement to inform external stakeholders of the value of commissioning for IPC and plans for the development of assurance framework and key IPC indicators. As emerging guidance regarding the NHS commissioning landscape came to the fore, in 2011, priority was given to the development of additional tools to support and inform members, these included an option appraisal and mapping documents, both of which outlined the function and future form of IPC in the new commissioning organisations. Following further consultation with members, the network produced the first IPC Commissioning Toolkit in March 2013 and this has been updated in March 2015. The toolkit provided commissioners with a set of IPC indicators for use in healthcare contracts. Additional resources have also been developed including two briefing paper documents which aimed to highlight the challenges regarding the changes within the new commissioning system and the potential impact on commissioning safe services with respect to IPC. These documents were released prior to the implementation of the changes in October 2013 and it was revised following the changes in March 2015.

## Results

The evaluation of the resources was positive, many alluded to the usefulness of the documents during a challenging period of uncertainty and used them to inform local decisions. There was also evidence that the toolkit had been used by commissioners and also providers in determining key performance indicators for IPC.

## Conclusion

Following the positive evaluation of the commissioning toolkit, there are plans to develop a third edition which will encompass various health care settings and social care.

## Disclosure of interest

None declared.

